# How neighborhood socioeconomic status, green space, and walkability are associated with risk for fracture among postmenopausal women

**DOI:** 10.1093/jbmrpl/ziaf024

**Published:** 2025-02-04

**Authors:** Marilyn E Wende, Matthew C Lohman, Daniela B Friedman, Alexander C McLain, Eric A Whitsel, Carolyn J Crandall, Jane A Cauley, Matthew Allison, Aladdin H Shadyab, Shawnita Sealy-Jefferson, Lorena Garcia, Michael B Cannell, Andrew T Kaczynski

**Affiliations:** Department of Health Education and Behavior, College of Health and Human Performance, University of Florida, Gainesville, FL 32611, United States; Department of Epidemiology and Biostatistics, Arnold School of Public Health, University of South Carolina, Columbia, SC 29208, United States; Department of Health Promotion, Education, and Behavior, Arnold School of Public Health, University of South Carolina, Columbia, SC 29201, United States; Department of Epidemiology and Biostatistics, Arnold School of Public Health, University of South Carolina, Columbia, SC 29208, United States; Department of Epidemiology, Gillings School of Global Public Health and Department of Medicine, School of Medicine, University of North Carolina at Chapel Hill, Chapel Hill, NC, 27516, United States; Division of General Internal Medicine and Health Services Research, Department of Medicine, David Geffen School of Medicine at University of California in Los Angeles, Los Angeles, CA 90095, United States; Department of Epidemiology, School of Public Health, University of Pittsburgh, Pittsburgh, PA 15261, United States; Division of Preventive Medicine, Department of Family Medicine, University of California San Diego, La Jolla, CA 92093, United States; Herbert Wertheim School of Public Health and Human Longevity Science, University of California, San Diego, La Jolla, CA 92093, United States; Department of Epidemiology, College of Public Health, The Ohio State University, Columbus, OH 43210, United States; Department of Public Health Sciences, School of Medicine, University of California Davis, Davis, CA 95616, United States; Department of Management, Policy & Community Health, School of Public Health, University of Texas Health Science Center in Houston, Dallas, TX 75207, United States; Department of Health Promotion, Education, and Behavior, Arnold School of Public Health, University of South Carolina, Columbia, SC 29201, United States; Prevention Research Center, Arnold School of Public Health, University of South Carolina, Columbia, SC 29208, United States

**Keywords:** neighborhood, socioeconomic status, walkability, green space, fracture, fracture prevention, fracture risk assessment, postmenopausal

## Abstract

Although most fractures, and about half of hip fractures, occur outdoors among older women, limited research has uncovered neighborhood predictors for fractures among older women. This study assessed the independent associations of neighborhood socioeconomic status (SES), walkability, and green space with incident any and hip fracture among postmenopausal women. The Women’s Health Initiative recruited a national sample of postmenopausal women (50-79 yr) across 40 U.S. clinical centers and conducted yearly assessments from 1993 to 2012 (*n* = 161 808). Women reporting a history of hip fracture or walking limitations were excluded from the analytic sample, yielding a final sample of 157 583 participants. Fracture events were self-reported and adjudicated annually. Walkability was calculated annually using measures of population density, land use mix, and presence/quantity of nearby high-traffic roadways. Neighborhood green space was calculated annually using measures of exposure to trees/vegetation. Neighborhood SES, walkability, and green space were categorized into tertiles: high, intermediate, and low. The time-varying relationship between neighborhood environmental factors and age at first fracture (any; hip) was examined using extended Cox proportional hazards modeling with adjustment. Neighborhood SES (intermediate vs low: hazard ratio = 1.03, 95% CI, 1.01-1.05; high vs low, hazard ratio = 1.01, 95% CI, 0.99-1.03) and green space (intermediate vs low, hazard ratio = 1.15, 95% CI, 1.12-1.18; high vs low hazard ratio = 1.18, 95% CI, 1.15-1.21) were associated with increased any incident fractures, while walkability had a mixed association (intermediate vs low hazard ratio = 1.06, 95% CI, 1.04-1.07; high vs low, hazard ratio = 0.97, 95% CI, 0.95-0.98). Neighborhood SES, walkability, and green space did not have a relationship with hip fracture after adjustment for important covariates. Results indicate that macroscale neighborhood features did not protect against fractures. Additional research is needed to investigate more granual neighborhood features that might influence injury risk and support physical activity among postmenopausal women.

## Introduction

Fracture events among adults 65 yr and older are projected to increase from 2.1 million in 2005 to over 3 million in 2025.^1^ Approximately 71% of fractures are reported by women, which makes up 75% of fracture-related health costs.^1^ Annual fracture incidence is greater than incidence of breast cancer and cardiovascular disease in women.^2^ This has health implications, as fractures can lead to permanent disability, loss of independence, and increased morbidity and mortality risk.^3^ Hip fracture (accounting for 14% of fractures)^1^ has especially devastating consequences, including decreased physical functioning, quality of life, social integration, independence, and overall health status, and increased mortality risk.^4^

Physical activity (PA) protects against fractures in older adults by improving bone density, enhancing balance and coordination, strengthening muscles, increasing mobility and function, promoting joint health, and improving reaction time.^5^^,^^6^ Most older women participate in PA in the form of walking outdoors.^7^^,^^8^ For this reason, emerging *Active Aging* initiatives focus on neighborhood barriers to aging and PA.^9^ In this context, older women are dependent on their neighborhood environments for PA and social interaction, and this has implications for health and well-being. Understanding neighborhood influences related to PA and their relationships with fracture are important since 65% of fractures (excluding hip and spine fractures) take place outdoors and hip fractures occur equally indoors and outdoors among older women.^10^

While the relationship between neighborhood attributes and fracture is understudied, some research has examined neighborhood socioeconomic status (NSES) in relation to hip fracture, and findings are mixed.^11–15^ NSES is most often measured using dimensions of income and/or wealth, education, and occupation.^16–20^ Some research shows that the relationship between NSES and fracture is more pronounced among older women compared to men, with higher NSES relating to lower odds of fracture.^11^^,^^13^^,^^14^ In addition, most research on this topic has taken place outside the United States,^11^^,^^13–15^ and research within the United States does not report results specific to older women.^12^

Other environmental features that may have a relationship with fracture risk among older women, such as walkability and green space, are understudied.^21^ Research shows that higher NSES areas often have increased walkability and green space,^22^^,^^23^ which facilitates PA.^24^^,^^25^ Walkability measures how supportive for walking and safe a space is, and has been shown to increase neighborhood walking and overall PA among older women.^26^^,^^27^ Green space, conceptualized as areas partially or fully covered by trees, grass, or other vegetation, has a beneficial impact on walking, as well as physical and mental health, among older adults.^28–30^ While green space provides safe, accessible, and aesthetically pleasing environments for outdoor PA, older women may also report safety or accessibility concerns in green spaces that can impact patterns of use.^31^

Current research on environmental fracture determinants primarily has taken place outside the United States and has not examined walkability and green space as protectants against fracture for community-dwelling older women.^11–15^ As fracture becomes an increasingly important public health issue in the United States, research is needed on whether fractures are associated with specific outdoor and environmental features, and how individual-level factors like walking levels, physical functioning, falling history, hormone therapy, body mass index, and rural residence, play a role. The Women’s Health Initiative (WHI) study and the related data allow researchers to uncover outdoor and environmental risk factors for fracture in women, given WHI is well-characterized on individual-level risk factors (eg, age, race, ethnicity, falling history), with large national reach (24 states, 40 clinical centers) and large sample size (nearly 162 000 participants) available to produce risk estimates.^32^ Moreover, the WHI study includes clinical trials, which allows researchers to examine whether involvement in hormone therapy, dietary modification, and calcium/vitamin D clinical trial study arms, as well as the observational study (OS), may modify the relationship between neighborhood exposures and fracture risk.^32^

The purpose of this current study was to identify understudied outdoor and neighborhood environmental features, including NSES, walkability, and green space, related to fracture occurrence in a large, diverse cohort of older women residing in community settings. We hypothesize that NSES, walkability, and green space will have a significant, negative impact on fall incidence.

## Materials and methods

### Study setting and sample

This study used data from the WHI, which enrolled women at 40 U.S. clinical centers from 1993 to 1998 (full details published elsewhere).^33^ Eligible women were aged 50-79 yr, postmenopausal, without medical conditions associated with a predicted survival of less than 3 yr, and planning to reside in the same geographic area for at least 3 yr.^34^^,^^35^ WHI study participants were followed and measured over time up to the present day, although observations will only be used up to 2012 because neighborhood exposures could only be ascertained up to this point. This study included data from both the WHI OS (*n* = 93 676) and the WHI clinical trials (CT; *n* = 68 132). Women reporting a history of hip fracture (*n* = 1404), high limitations with walking a block or more at baseline (*n* = 2891), or both (*n* = 70) were excluded, yielding a final sample of 157 583 participants.

WHI CT participants were randomized into 1 or more of 3 randomized controlled trials: dietary modification, hormone therapy, and calcium/vitamin D supplementation (described elsewhere).^33^ Women could also choose to take part in the OS, where women provided information about their medical histories and health habits. Baseline characteristics of OS and CT participants are published elsewhere.^34^ Institutional review board approval was obtained at each clinical center and all participants provided written informed consent.

### Data collection

Demographic, behavioral, and medical history data were obtained by self-report using standardized questionnaires at baseline. Participants were asked to report residential addresses at baseline, which were regularly updated throughout follow-up and accurately geocoded using ArcGIS 10.2.2 software.

### Participant measures

Fracture events (any, hip) were collected annually through 2012. Participants were asked “Since [last reporting date], has a doctor told you that you had a broken, fractured, or crushed bone?”, and then specified the fracture location. Hip fractures were centrally adjudicated using radiographic or operative reports during the main study and extension 1 for the CT (1993-2010). Hip fractures were self-reported for extension 2 (2010-2014). All other types of fractures were locally adjudicated (1993-2005), and then only self-reported (2005-2012). Any fractures included all reported clinical fractures (including hip fracture) except for those of the ribs, sternum, skull, face, fingers, toes, and cervical vertebrae.

Age at baseline (categorized as <50-59, 60-69, 70-79+ yr), household income level at baseline (categorized as *<*$34 999, $35 000-$74 999, *>*$75 000), race and ethnicity (categorized as Non-Hispanic White, Black/African American, Hispanic/Latino, Asian or Pacific Islander, American Indian or Alaska Native, Other), vision impairment at baseline (categorized as none, mild, moderate, severe), corticosteroid use at baseline (categorized as yes or no), bone medication use at baseline (including Fosamax, Actonel, Boniva, Reclast, and Miacalcin; categorized as yes or no), and marital status at baseline (categorized as presently married, divorced/separated, widowed, never married, marriage-like relationship) were obtained by self-report using enrollment questionnaires. Walking in metabolic equivalent of task-hours per week (MET-hr/wk) was based on the self-reported type, duration, and frequency, then grouped according to tertiles (none, low, intermediate, high) for more meaningful interpretation. Presence or absence of physical functioning limitations was self-reported at baseline using indicators of health and general functioning. This measure is correlated with frailty indicators: walking speed (*r* = −0.34) and grip strength (*r* = 0.14).^36^

At baseline, frequency of falling history within the previous year was self-reported, excluding falls due to sports activities. Hormone therapy use (categorized as never used, past user, current user) was self-reported at baseline and included any female hormones (ie, estrogen, progesterone). Body mass index (underweight, normal weight, overweight, obese classes 1, 2, 3) was assessed at baseline, where certified staff took height and weight measurements at clinic visits.

### Neighborhood measures

Participants reported residential addresses at baseline and throughout follow-up, which were geocoded. To avoid errors, geographic procedures relied on geocoded address points (coordinates) in polygon joins with neighborhood polygons. Geocoded participant addresses were assigned to rural–urban commuting area codes of their respective Census tracts (from U.S. Department of Agriculture and Economic Research Service) were used to estimate rurality and were categorized as urban, large rural city/town, small rural town, and isolated small, rural town.

NSES included dimensions of income, wealth, education, and occupation. Income was measured using median household income; percentage of households with interest, dividends/rent income; and median value of owner-occupied housing. Education was the percentage of adults with high school and college degrees. Occupation was the percentage of adults with professional, managerial, or executive occupations. Census tract-level data for all measures were collected from the U.S. Census of Population and Housing for 2000 and American Community Survey for 2005-2009 using 5-yr estimates and merged with participant-level data using census tract identifiers that most closely corresponded with the year data was collected (SAS 9.4 software). NSES was calculated by natural log-transforming the median household income and median value of housing units, z-transforming all 6 dimensions, and summing them to create a summary z-score, separately for OS and CT participants annually. Z-scores were categorized into tertiles (low, intermediate, high) to provide more meaningful interpretations.

Neighborhood walkability was measured based on the “3 D’s” (ie, density, diversity, and design) associated with walking.^37^ Density was measured using U.S. Census of Population and Housing attributes data from 1993 to 2012, as the ratio of population count to land area (ie, persons per kilometer^2^) within a geocoded participant address-centric Euclidean buffer of 5.0 miles (calculated using ArcGIS 10.2.2 software). Diversity was measured using Euclidean areal measures of exposure to developed land and cross-walked over National Land Cover Databases (NLCD). NLCD data were used from 1992, 2001, 2006, and 2011 to create 500-m geocoded participant address-centric Euclidean buffers of land cover types (ie, water, developed, barren, forest, shrubland, herbaceous, planted/cultivated, wetlands) using ArcGIS 10.2.2 software. The Herfindahl–Hirschman Index was used to assess diversity of land cover.^38^ Design (ie, exposure to high traffic roadways) was based on ESRI mapped and Census feature class coded A1-A3 roadways (annual Street Map data 1993-2000, 2003, ESRI & Maps data 2001-2002, 2003, 2004, 2005, 2006, 2007-2009, 2010-2012). A1-A3 roadways include interstate/toll highways with interchanges, national and regional highways without limited access, state highways, and some county highways that connect smaller towns, subdivisions, and neighborhoods. Using ArcGIS 10.2.2 software, geocoded participant address-centric Euclidean buffers of 500 m were employed to identify the total length in meters of A1-A3 roads within the empirically supported distances. Road lengths were summed to estimate total meters of A1-A3 roadways proximal to participant addresses annually (1993-2012). Walkability components were standardized separately (PROC STANDARD, SAS 9.4 software) then averaged to create composite scores that were categorized into tertiles (low, intermediate, high) to provide more meaningful interpretations.

Neighborhood green space was ascertained using Euclidean areal measures of exposure to trees/vegetation that were cross-walked over NLCD. Similar to the diversity calculation, NLCD data (from 1992, 2001, 2006, and 2011) were used to create 500-m geocoded participant address-centric Euclidean buffers of land cover types (ie, water, developed, barren, forest, shrubland, herbaceous, planted/cultivated, wetlands) using ArcGIS 10.2.2 software. As in past green space research,^25^^,^^39^ fractions of the total buffer area (in m^2^) occupied by each land cover type determined the areal percentage of vegetative land types, including forest, shrubland, planted/cultivated, and herbaceous grasslands/wetlands. Estimates were standardized (PROC STANDARD, SAS 9.4 software) and converted into tertiles (low, intermediate, high) to provide more meaningful interpretations.

Data on NSES, walkability, and green space were merged with annual fall data according to year(s) since enrollment. For years with missing data on environmental exposures, it was assumed that the environmental exposure did not change since the most recent data collection time point with available data. Large buffer sizes were used to minimize missingness for walkability and green space exposures (since NSES measures were only available on the census tract level), so sensitivity analyses were completed using smaller (ie, 100-m buffer for green space, diversity of land cover, and exposure to A1-A3 highways, 0.75-mile buff for population density) buffers to justify our approach.

### Statistical analyses

Descriptive information and crude relationships for exposures and outcomes of interest are presented using Pearson’s chi-square tests for nominal categorical variables and Spearman Rank tests of correlation for ordinal categorical variables. Next, we examined the time-varying relationship between neighborhood environmental factors and age at first fracture (any; hip) using extended Cox proportional hazards modeling.^40^ Windows of time since enrollment (where environmental variables were collected) were age-standardized to adjust for the different time scales.^41^ To estimate the association between neighborhood characteristics and time to first fracture, we first fit crude models for each independent neighborhood exposure. Models were then presented after adjustment for additional covariates. Adjustment variables, chosen *a priori*, included baseline income level, race and ethnicity, marital status, climate region, rurality, vision impairment, and WHI study arm assignment.

Interaction terms were chosen *a priori* and included study arm assignment, race and ethnicity, baseline walking levels, baseline low physical functioning, baseline fall history, baseline hormone therapy use, baseline body mass index, and rurality. If effect modification was found based on product terms for the interaction (*α* = 0.01, Bonferroni-corrected threshold), we presented models stratified on these variables.

As stated previously, analyses for neighborhood walkability and green space and their crude and adjusted relationships with any fractures and hip fracture were replicated using smaller (100 vs 500 m in original analyses) buffers. All analyses were completed using SAS 9.4 software.

## Results

### Sample characteristics

Table 1 describes the sample characteristics of study participants (*N*= 157 583), and their cross-sectional relationships with *any clinical* fracture and *hip* fracture. At baseline, the highest percentage of participants had no history of falling in the year prior to baseline (64.9%), were 60-69 yr of age (44.9%), were considered normal weight (34.2%) or overweight (34.6%), identified as White (85.5%), not Hispanic/Latino/Spanish (95.5%), were married (60.7%), had never used hormone therapy (43.6%), had no trouble with vision (80.8%), had income of <$34 999 (37.9%) or between $35 000 and $74 999 (37.9%), reported at least some walking (19.5% low, 25.1% intermediate, 20.2% high), and did not report low physical functioning (69.4%). In addition, the highest percentage of participants lived in the Northeast climate region (22.8%) and in urban locations (82.0%). The Appendix provides information on the relationships between NSES, walkability, and green space at baseline.

**Table 1:** Demographic and physiologic characteristics at baseline and fracture outcomes from 1993 to 2012 (*N* = 157 583).

**Variable**	**Overall**	**Any fracture** ^**1**^	**No fracture**	***Χ*** ^**2**^ **statistic**	**Any hip fracture** ^**1**^	**No hip fracture**	***Χ*** ^**2**^ **statistic**
	# (%)	# (%)	n (%)	*p*-value^2^	n (%)	n (%)	(*p*-value)^2^
**Total**	157 583 (100)	63 641 (40.4)	93 942 (59.6)	–	7093 (4.5)	150 490 (95.5)	–
**Socioeconomic status** ^**3**^							
**Missing**	96 (0.1)	10 (10.4)	86 (89.6)	**648.4 (<.0001)**	2 (2.3)	84 (97.7)	**57.7 (<.0001)**
**Low**	52 448 (33.3)	18 970 (36.2)	33 478 (63.8)		2102 (4.0)	50 346 (96.0)	
**Intermediate**	52 543 (33.3)	21 708 (41.3)	30 835 (58.7)		2374 (4.5)	50 169 (95.5)	
**High**	52 496 (33.3)	22 951 (43.7)	29 545 (56.3)		2614 (5.0)	49 882 (95.0)	
**Walkability** ^**3**^							
**Missing**	87 (0.1)	26 (29.9)	61 (70.1)	**19.5 (<.0001)**	3 (3.4)	85 (96.6)	**7.9 (0.0189)**
**Low**	52 483 (33.3)	21 513 (41.0)	30 970 (59.0)		2471 (4.7)	50 012 (95.3)	
**Intermediate**	52 478 (33.3)	21 273 (40.5)	31 205 (59.5)		2323 (4.4)	50 155 (95.6)	
**High**	52 535 (33.3)	20 843 (39.7)	31 692 (60.3)		2297 (4.4)	50 238 (95.6)	
**Green space** ^**3**^							
**Missing**	3308 (2.10)	939 (28.4)	2369 (71.6)	**154.6 (<.0001)**	67 (2.0)	3241 (98.0)	**26.0 (<.0001)**
**Low**	51 547 (32.7)	19 821 (38.5)	31 726 (61.5)		2169 (4.2)	49 378 (95.8)	
**Intermediate**	50 906 (32.3)	21 144 (41.5)	29 762 (58.5)		2479 (4.8)	48 427 (95.1)	
**High**	51 822 (32.9)	21 726 (41.9)	30 096 (58.1)		2376 (4.6)	49 446 (95.4)	
**Study arm**							
**Dietary clinical trial arm**	9659 (6.1)	3745 (38.8)	5914 (61.2)	**106.8 (<.0001)**	426 (4.4)	9233 (95.6)	7.5 (0.1836)
**Hormone therapy clinical trial arm (estrogen + progesterone)**	2811 (1.8)	1167 (41.5)	1644 (58.5)		148 (5.3)	2663 (94.7)	
**Hormone therapy clinical trial arm (estrogen alone)**	2001 (1.3)	783 (39.1)	1218 (60.9)		90 (4.5)	1911 (95.5)	
**Calcium/Vitamin D clinical trial arm**	35 542 (22.5)	15 164 (42.7)	20 378 (57.3)		1646 (4.6)	33 896 (95.4)	
**Clinical trial control group**	16 528 (10.5)	6566 (39.7)	9962 (60.3)		706 (4.3)	15 822 (95.7)	
**Observational study arm**	91 042 (57.8)	36 216 (39.8)	54 826 (60.2)		4077 (4.5)	86 965 (95.5)	
**Fall history at baseline**							
**Missing**	6485 (4.1)	2684 (41.4)	3801 (58.6)	**1006.5 (<.0001)**	311 (4.8)	6174 (95.2)	**55.7 (<.0001)**
**None**	102 294 (64.9)	38 704 (37.8)	63 590 (62.2)		4332 (4.2)	97 962 (95.8)	
**1 time**	30 252 (19.2)	13 145 (43.4)	17 107 (56.6)		1457 (4.8)	28 795 (95.2)	
**2 times**	12 449 (7.9)	5947 (47.8)	6502 (52.2)		657 (5.3)	11 792 (94.7)	
**3 or more times**	6103 (3.9)	3161 (51.8)	2942 (48.2)		336 (5.5)	5767 (94.5)	
**Age, yr**							
**<50-59**	52 614 (33.4)	19 590 (37.2)	33 024 (62.8)	**363.7 (<.0001)**	948 (1.8)	51 666 (98.2)	**2145.6 (<.0001)**
**60-69**	70 835 (44.9)	29 269 (41.3)	41 566 (58.7)		3253 (4.6)	67 582 (95.4)	
**70-79+**	34 134 (21.7)	14 782 (43.3)	19 352 (56.7)		2892 (8.5)	31 242 (91.5)	
**Race**							
**Missing/unspecified**	3140 (2.0)	690 (22.0)	2450 (78.0)	**3078.9 (<.0001)**	41 (1.3)	3099 (98.7)	**668.7 (<.0001)**
**White**	134 688 (85.5)	58 097 (43.1)	76 591 (56.9)		6798 (5.0)	127 890 (95.0)	
**Black or African American**	13 711 (8.7)	3029 (22.1)	10 682 (77.9)		123 (0.9)	13 588 (99.1)	
**American Indian or Alaska Native**	515 (0.3)	167 (32.4)	348 (67.6)		5 (1.0)	510 (99.0)	
**Asian**	4027 (2.6)	1107 (27.5)	2920 (72.5)		76 (1.9)	3951 (98.1)	
**Pacific Islander**	147 (0.1)	38 (25.9)	109 (74.1)		1 (0.7)	146 (99.3)	
**Two or more**	1355 (0.9)	513 (37.9)	842 (62.1)		49 (3.6)	1306 (96.4)	
**Ethnicity**							
**Missing/unspecified**	1480 (0.9)	195 (13.2)	1285 (86.8)	**616.0 (<.0001)**	6 (0.4)	1474 (99.6)	**182.7 (<.0001)**
**Not Hispanic/Latino/Spanish**	150 530 (95.5)	61 622 (40.9)	88 908 (59.1)		7002 (4.7)	143 528 (95.3)	
**Puerto Rican**	756 (0.5)	250 (33.1)	506 (66.9)		13 (1.7)	743 (98.3)	
**Mexican, Mexican American Chicana**	2606 (1.7)	815 (31.3)	1791 (68.7)		30 (1.2)	2576 (98.8)	
**Cuban**	385 (0.2)	143 (37.1)	242 (62.9)		10 (2.6)	375 (97.4)	
**Other**	1826 (1.2)	616 (33.7)	1210 (66.3)		32 (1.7)	1794 (98.3)	
**Marital status at baseline**							
**Missing**	741 (0.5)	230 (31.0)	511 (69.0)	**115.4 (<.0001)**	28 (3.8)	713 (96.2)	**261.7 (<.0001)**
**Presently married**	95 631 (60.7)	39 430 (41.2)	56 201 (58.8)		4192 (4.4)	91 439 (95.6)	
**Divorced or separated**	25 003 (15.9)	9400 (37.6)	15 603 (62.4)		831 (3.3)	24 172 (96.7)	
**Widowed**	26 770 (17.0)	10 818 (40.4)	15 952 (59.6)		1653 (6.2)	25 117 (93.8)	
**Never married**	6879 (4.3)	2694 (39.2)	4185 (60.8)		290 (4.2)	6589 (95.8)	
**Marriage-like relationship**	2559 (1.6)	1069 (41.8)	1490 (58.2)		99 (3.9)	2460 (96.1)	
**Hormone therapy use at baseline**							
**Missing**	136 (0.1)	52 (38.2)	84 (61.8)	**51.7 (<.0001)**	8 (5.9)	128 (94.1)	**71.8 (<.0001)**
**Never used**	68 660 (43.6)	27 128 (39.5)	41 532 (60.5)		3291 (4.8)	65 369 (95.2)	
**Past user**	25 186 (16.0)	10 579 (42.0)	14 607 (58.0)		1268 (5.0)	23 918 (95.0)	
**Current user**	63 601 (40.3)	25 882 (40.7)	37 719 (59.3)		2526 (4.0)	61 075 (96.0)	
**Trouble with vision at baseline**							
**Missing**	1676 (1.1)	574 (34.3)	1102 (65.7)	**14.1 (.0028)**	64 (3.8)	1612 (96.2)	4.3 (.2313)
**None**	127 388 (80.8)	51 370 (40.3)	76 018 (59.7)		5703 (4.5)	121 685 (95.5)	
**Mild**	20 770 (13.2)	8534 (41.1)	12 236 (58.9)		986 (4.7)	19 784 (95.3)	
**Moderate**	6161 (3.9)	2568 (41.7)	3593 (58.3)		263 (4.3)	5898 (95.7)	
**Severe**	1588 (1.0)	595 (37.5)	993 (62.5)		77 (4.8)	1511 (95.2)	
**Baseline household income**							**56.4 (<.0001)**
**Missing**	10 486 (6.7)	3857 (36.8)	6629 (63.2)	**281.4 (<.0001)**	453 (4.3)	10 033 (95.7)	
***<*$34 999**	59 711 (37.9)	22 946 (38.4)	36 765 (61.6)		2939 (4.9)	56 772 (95.1)	
**$35 000-$74 999**	59 724 (37.9)	24 906 (41.7)	34 818 (58.3)		2646 (4.4)	57 078 (95.6)	
***>*$75 000**	27 662 (17.5)	11 932 (43.1)	15 730 (56.9)		1055 (3.8)	26 607 (96.2)	
**Walking levels (MET-hr/wk) at baseline**				**54.6 (<.0001)**			**8.9 (0.0303)**
**Missing**	7348 (4.7)	3188 (43.4)	4160 (56.6)		350 (4.8)	6998 (95.2)	
**None (0 MET-hr/wk)**	48 212 (30.5)	18 767 (38.9)	29 445 (61.1)		2086 (4.3)	46 126 (95.7)	
**Low (0-6.8 MET-hr/wk)**	30 650 (19.5)	12 391 (40.4)	18 259 (59.6)		1390 (4.5)	29 260 (95.5)	
**Intermediate (6.8-16.7 MET-hr/wk)**	39 577 (25.1)	16 210 (41.0)	23 367 (59.0)		1870 (4.7)	37 707 (95.3)	
**High (16.7+ MET-hr/wk)**	31 796 (20.2)	13 085 (41.2)	18 711 (58.8)		1397 (4.4)	30 399 (95.6)	
**Low physical functioning**							
**Missing**	3003 (1.9)	1033 (34.4)	1970 (65.6)	**5.1 (.0240)**	123 (4.1)	2880 (95.9)	**33.6 (<.0001)**
**Yes**	45 281 (28.7)	18 538 (40.9)	26 743 (59.1)		2257 (5.0)	43 024 (95.0)	
**No**	109 299 (69.4)	44 070 (40.3)	65 229 (59.7)		4713 (4.3)	104 586 (95.7)	
**Climate region**							
**Northeast**	35 922 (22.8)	15 611 (43.5)	20 311 (56.5)	**455.8 (<.0001)**	1833 (5.1)	34 089 (94.9)	**100.1 (<.0001)**
**South–lower**	26 251 (16.7)	9548 (36.4)	16 703 (63.6)		1041 (4.0)	25 210 (96.0)	
**South–upper**	14 442 (9.2)	5858 (40.6)	8584 (59.4)		584 (4.0)	13 858 (96.0)	
**Midwest–lower**	7272 (4.6)	3234 (44.5)	4038 (55.5)		367 (5.1)	6905 (94.9)	
**Midwest–upper**	27 460 (17.4)	10 987 (40.0)	16 473 (60.0)		1163 (4.2)	26 297 (95.8)	
**West–lower**	23 535 (14.9)	8898 (37.8)	14 637 (62.2)		939 (4.0)	22 596 (96.0)	
**West–middle**	15 596 (9.9)	6570 (42.1)	9026 (57.9)		806 (5.2)	14 790 (94.8)	
**West–upper**	7105 (4.5)	2935 (41.3)	4170 (58.7)		360 (5.1)	6745 (94.9)	
**Rurality**							
**Missing**	18 480 (11.7)	7193 (38.9)	11 287 (61.1)	2.0 (.5766)	782 (4.2)	17 698 (95.8)	3.2 (.3595)
**Urban**	129 177 (82.0)	52 359 (40.5)	76 818 (59.5)		5831 (4.5)	123 346 (95.5)	
**Large rural city/town**	5570 (3.5)	2303 (41.4)	3267 (58.6)		261 (4.7)	5309 (95.3)	
**Small, rural town**	2388 (1.5)	986 (41.3)	1402 (58.7)		116 (4.9)	2272 (95.1)	
**Isolated, small rural town**	1968 (1.3)	800 (40.6)	1168 (59.4)		103 (5.2)	1865 (94.8)	

Abbreviation: MET-hr/wk, metabolic equivalent of task-hours per week.

^1^Variables relate to whether the participant experiences a fracture (any or hip) over the entire study period (1993-2012).

^2^Pearson’s chi-square tests were used for nominal categorical predictors and Spearman Rank tests were used for ordinal categorical predictors.

^3^Neighborhood socioeconomic status, walkability, and green space tertile were calculated according to each participant’s measurement at enrollment.

### Neighborhood socioeconomic status and fracture

Table 2 describes the relationships between NSES, walkability, and green space with incident fracture (any and hip). NSES had a minimal association with any fracture *after adjustment* for important covariates (intermediate vs low SES hazard ratio [HR] = 1.03, 95% CI, 1.01-1.05; high vs low SES HR = 1.01, 95% CI, 0.99-1.03), with intermediate compared to low SES being associated with hazard of any fracture (Table 2). Of the interaction terms tested (study arm assignment, race and ethnicity, baseline walking levels, baseline low physical functioning, baseline fall history, baseline hormone therapy, baseline body mass index, and rurality), none were significant (*α* = .01).

**Table 2:** Cox regression analysis of neighborhood and demographic characteristics on fracture incidence, 1993-2012 (*N* = 157 583).

	**Any fracture**			**Hip fracture**		
	**HR**	**95% CI**	***p*-value**	**HR**	**95% CI**	***p-*value**
**Model—NSES**						
**Model 1a (Unadjusted)^1^**						
**Intermediate SES**	1.06	(1.04, 1.08)	**<.0001**	1.10	(1.04, 1.16)	**.0015**
**High SES**	1.06	(1.04, 1.08)	**<.0001**	1.02	(0.97, 1.08)	.4088
**Model 2a (model 1a + confounders)^1^^,^^2^**						
**Intermediate SES**	1.03	(1.01, 1.05)	**0.0018**	1.04	(0.98, 1.10)	.2320
**High SES**	1.01	(0.99, 1.03)	0.1727	0.95	(0.90, 1.01)	.0754
**Model—Neighborhood walkability**						
**Model 1b (Unadjusted)^3^**						
**Intermediate walkability**	1.04	(1.02, 1.06)	**<.0001**	1.03	(0.98, 1.09)	.2320
**High walkability**	1.03	(1.02, 1.05)	**<.0001**	1.03	(0.98, 1.08)	.3084
**Model 2b (model 1b + confounders)^2^^,^^3^**						
**Intermediate walkability**	1.06	(1.04, 1.07)	**<.0001**	1.01	(0.96, 1.07)	.6269
**High walkability**	0.97	(0.95, 0.98)	**<.0001**	0.95	(0.90, 1.00)	.0675
**Model—Neighborhood green space**						
**Model 1c (Unadjusted)^4^**						
**Intermediate green space**	1.10	(1.07, 1.12)	**<.0001**	1.06	(0.98, 1.14)	.1552
**High green space**	1.14	(1.12, 1.17)	**<.0001**	1.11	(1.02, 1.20)	**.0117**
**Model 2c (model 1c + confounders)^2^^,^^4^**						
**Intermediate green space**	1.15	(1.12, 1.18)	**<.0001**	1.03	(0.96, 1.12)	.4021
**High green space**	1.18	(1.15, 1.21)	**<.0001**	1.05	(0.96, 1.14)	.2704

Abbreviations: HR, hazard ratio; NSES, neighborhood socioeconomic status; SES, socioeconomic status.

Bolded numbers represent significance with *p* < .05, and *p* < .01 for interaction terms (to account for Bonferonni correction).

*p*-values for interaction terms represent joint tests of association. Joint tests for an effect test that all the parameters associated with that effect are 0.

^1^Referent category: low NSES.

^2^Adjusted for age, household income, race, ethnicity, corticosteroid use, bone medication use, marital status, climate region, rurality, vision impairment, and study arm.

^3^Referent category: low walkability.

^4^Referent category: low green space.

NSES did not have a significant association with hip fracture *after adjustment* for important covariates (Table 2). Of the interaction terms tested (study arm assignment, race and ethnicity, baseline walking levels, baseline low physical functioning, baseline fall history, baseline hormone therapy, baseline body mass index, and rurality), none were significant (*α* = .01).

### Neighborhood walkability and fracture

Neighborhood walkability was significantly associated with any fracture *after adjustment* for important covariates (Table 2). The HRs indicate a modestly increased risk of any fracture for intermediate walkability (HR = 1.06, 95% CI, 1.04-1.07) and decreased risk of any fracture for high walkability (HR = 0.97, 95% CI, 0.95-0.98), both compared to low walkability. Of the interaction terms tested (study arm assignment, race and ethnicity, baseline walking levels, baseline low physical functioning, baseline fall history, baseline hormone therapy, baseline body mass index, and rurality), none were significant (*α* = .01).

Neighborhood walkability did not have a relationship with *hip* fracture after adjustment for important covariates (Table 2). Of the interaction terms tested (study arm assignment, race and ethnicity, baseline walking levels, baseline low physical functioning, baseline fall history, baseline hormone therapy, baseline body mass index, and rurality), none were significant (*α* = .01).

### Neighborhood green space and fracture

Neighborhood green space had a relationship with *any* fracture after adjustment for important covariates (Table 2). The HRs demonstrated an increased risk of *any* fracture for intermediate green space (HR = 1.15, 95% CI, 1.12-1.18) and high green space (HR = 1.18, 95% CI, 1.15-1.21), both compared to low green space. Of the interaction terms tested (study arm assignment, race and ethnicity, baseline walking levels, baseline low physical functioning, baseline fall history, baseline hormone therapy, baseline body mass index, and rurality), none were significant (*α* = .01).

Neighborhood green space did not have a relationship with *hip* fracture after adjustment for important covariates (Table 2). Of the interaction terms tested (study arm assignment, race and ethnicity, baseline walking levels, baseline low physical functioning, baseline fall history, baseline hormone therapy, baseline body mass index, and rurality), none were significant (*α* = .01).

### Sensitivity analyses

Table 3 presents results of sensitivity analyses using smaller buffer sizes (ie, 100-m buffer for green space, diversity of land cover, and exposure to A1-A3 highways, 0.75-mile buffer for population density) for the relationships between walkability, green space, and fracture (any and hip). For walkability, sensitivity analyses showed that high compared to low walkability had 10% lower hazards for *any* fracture (HR = 0.90, 95% CI, 0.88-0.93, compared to only 3% lower hazards for hip fracture between high and low walkability in the original analyses) after adjustment. The relationship between walkability and *any* fracture was no longer significant in a comparison of intermediate to low walkability using smaller buffer sizes after adjustment. For green space, sensitivity analyses showed that intermediate compared to low green space had 22% higher hazards for *any* fracture (HR = 1.22, 95% CI, 1.20-1.24, compared to only 15% higher hazards for hip fracture between intermediate and low green space in the original analyses) after adjustment. The relationship between green space and *hip* fracture was significant with smaller buffer sizes, with high green space with 15% higher hazards (HR = 1.15, 95% CI, 1.04-1.28) and intermediate green space with 9% higher hazards (HR = 1.09, 95% CI, 1.01-1.17) for hip fracture compared to low green space after adjustment.

**Table 3:** Sensitivity analysis using reduced buffer sizes for the relationships between neighborhood walkability, green space, and fracture incidence, 1993-2012 (*N* = 157 583).

	**Any fracture**			**Hip fracture**		
	**HR**	**95% CI**	***p*-value**	**HR**	**95% CI**	***p*-value**
**Model—Neighborhood walkability**						
**Model 1b (Unadjusted)^1^**						
**Intermediate walkability**	1.02	(1.00, 1.04)	.1049	1.04	(0.98, 1.12)	.2189
**High walkability**	0.99	(0.97, 1.02)	.6041	1.01	(0.92, 1.10)	.8690
**Model 2b (model 1b + confounders)^1^^,^^2^**						
**Intermediate walkability**	0.98	(0.96, 1.00)	.0619	1.04	(0.97, 1.12)	.2814
**High walkability**	0.90	(0.88, 0.93)	**<.0001**	0.98	(0.89, 1.07)	.6207
**Model—Neighborhood green space**						
**Model 1c (Unadjusted)^3^**						
**Intermediate green space**	1.12	(1.10, 1.14)	**<.0001**	1.07	(1.00, 1.15)	.0546
**High green space**	1.15	(1.12, 1.18)	**<.0001**	1.19	(1.08, 1.32)	**.0006**
**Model 2c (model 1c + confounders)^2^^,^^3^**						
**Intermediate green space**	1.22	(1.20, 1.25)	**<.0001**	1.09	(1.01, 1.17)	**.0231**
**High green space**	1.18	(1.15, 1.21)	**<.0001**	1.15	(1.04. 1.28)	**.0079**

Abbreviation: HR, hazard ratio.

Bolded values represent significance with *p* < .05, and *p* < .10 for interaction terms (to account for Bonferroni correction).

*p*-values for interaction terms represent the joint tests of association. The joint test for an effect is a test that all the parameters associated with that effect are 0.

^1^Referent category: low walkability.

^2^Adjusted for age, household income, race & ethnicity, marital status, climate region, rurality, vision impairment, and study arm.

^3^Referent category: low green space.

## Discussion

### Summary of findings

Results from this study showed that NSES, walkability, and green space had very modest associations with any fracture incidence after adjustment for covariates. NSES, walkability, and green space had no relationship with hip fracture incidence after adjustment. Furthermore, study arm assignment, race and ethnicity, baseline walking levels, baseline low physical functioning, baseline fall history, baseline hormone therapy, baseline body mass index, and rurality did not modify the relationships between NSES, walkability, and green space and any and hip fracture. These results contribute to existing literature on neighborhood environments and risk of injury by identifying whether a longitudinal relationship between positive neighborhood attributes and any fracture and hip fracture exists in a national sample of community-dwelling older women. In addition, these results contribute to a growing body of research on features of neighborhood environments that may facilitate aging in place and maintaining functional independence.

### Comparison to prior research

Among postmenopausal women in the WHI residing in diverse locations of the United States, intermediate and high compared to low NSES showed modestly increased risk for *any* fracture after adjustment. The relationship between NSES and *hip* fracture, an outcome with detrimental health implications,^4^^,^^42^ was significant only before adjustment for important covariates. Past research on outdoor neighborhood risk factors for fracture has only examined the relationship between area-level SES and hip fracture, and results were mixed: some research reported a 6%-30% reduction in hip fracture risk when comparing high to low-income areas,^12–14^ while other studies reported that low-income areas showed reduced hip fracture risk compared to high income (with a 30% risk reduction in one study).^11^^,^^15^ Prior research that showed a major protective effect of lower SES on hip fracture while the excess risk of hip fracture among higher income groups was mostly attenuated after adjusting for age and sex differences.^15^ Another study that showed lower SES participants had decreased risk for hip fracture had mixed findings when stratified by gender: average compared to low SES had significantly increased odds and high compared to low SES had no significant difference in odds of hip fracture among women.^11^ Existing research showed major similarities with our findings after accounting for gender differences in their results.

This study showed that high walkability was associated with decreased risk and intermediate walkability was associated with increased risk, for *any* fracture when compared to low walkability, and walkability was not related to *hip* fracture after adjustment. The relationship between walkability and fracture risk has been understudied, although one previous study did show that perceptions of walkability were shaped by the health status of older adults,^43^ and another study that used objective environmental measures showed that increased walkability was protective against fracture among older men and women (with a stronger relationship among men compared to women).^21^ We did not find that low physical functioning or history of falling interacted with objectively-measured neighborhood walkability to impact any fracture incidence. Interestingly, sensitivity analyses using smaller buffer sizes showed that high compared to low walkability had a more pronounced relationship with *any* fracture compared to analyses using larger buffer sizes, and the direction of the relationship between intermediate walkability and *any* fracture changed. That is, there was a clear dose response relationship between neighborhood walkability and lower risk of fracture using smaller buffer sizes. Past research shows that older adults (79-94 yr old) in Finland reported interacting with walking hazards closer to home (median distance = 166 m) and neighborhood destinations further from home (median distance = 492 m).^44^ In addition, this study showed that neighborhood barriers and destinations increased the odds of PA only when they were located further than 500 m from their homes.^44^ Conversely, our study shows that proximal neighborhood environments do show a greater association with fracture incidence compared to analyses using larger neighborhood buffers. This may be explained by differences between Finland and the United States in terms of pedestrian environments and neighborhood supports for older adults, which must be explored further in future research.

The relationship between green space and fracture is also understudied, although one past study showed that objectively-measured residential greenness was associated with decreased incident fracture among older men and women.^21^ This relationship was strongest in neighborhoods with high walkability.^21^ Our study did find that higher compared to lower levels of green space were associated with higher any fracture risk among postmenopausal women. One reason for this finding may be that those with higher levels of green space near their home residence interact with their outdoor environments more often and maintain higher levels of PA, which may decrease risk for more serious hip fractures but also increase risk for any fracture.^29^^,^^45^ Despite this, our results showed that walking levels did not modify the relationships between neighborhood green space and *any* or *hip* fracture risk. Another possible explanation for our findings may be that areas with higher levels of green space may also have fewer community resources for older adults. While our measure for green space accurately captures the presence of vegetation, data on the presence of age-appropriate PA equipment and amenities (eg, benches, shade) and the absence of environmental hazards (eg, litter, uneven sidewalks) was not collected as part of this study.

### Implications for public health research

This study has important implications for public health research. First, we were able to establish modest, longitudinal associations between objectively measured NSES, walkability, and green space and fracture. Future research should examine more granular environmental factors that may influence fracture risk over time, such as air temperature, weather conditions (eg, precipitation, snow and ice), or safety factors such as traffic and sidewalks. Ecological analyses examining key environmental determinants may be useful for uncovering associations with population-level fracture outcomes. Additional research on environmental exposures can utilize existing population-based datasets, such as the American Academy of Orthopaedic Surgeons Fracture and Trauma registry. Future research can also use quasi-experimental study designs to assess if changes to neighborhood walkability and green spaces decrease fracture risk among older women, perhaps by improving environments in ways that promote engagement among older adults (eg, decreasing traffic speeds, supplementing green space with shaded areas). Quasi-experimental methods can also help uncover whether infrastructure improvements show efficacy for decreasing fracture risk among women living in areas with high levels of green space, since our results showed that increased green space significantly *increased* risk for any fracture. Lastly, researchers may explore if specific variables that did not interact with our environmental exposure to impact fracture, such as walking levels, body mass index, and physical impairment status, may mediate the relationships between NSES, walkability, and green space and fracture.

### Implications for public health practice

This research has several implications for public health practice. First, this study contributes to growing research and initiatives on *aging in place.* The World Health Organization (WHO)’s policy framework for *Active Aging* acknowledges the importance of environments for aging independently. Worldwide, the WHO and other organizations/partners are working to identify major physical and social barriers to aging in community settings*.* This study informs WHO initiatives to improve independent aging, by providing input on the role of outdoor environmental features, such as green spaces, walkability, and socioeconomic conditions. Second, this study can help identify geographic locations where postmenopausal women may be at the highest risk for fracture. This information can be used to improve policy, systems, and environmental change approaches for addressing gender disparities in PA and fracture outcomes and increase the chances that women can age independently in their homes. Adding to this, we collected objective measurements of neighborhood environments. Practitioners can use similar methods to more easily identify locations that need intervention using publicly available administrative data that is objective. It is feasible for local practitioners to map rates of fracture among older women using administrative data and identify whether areas with higher rates of fracture also have poor environmental supports (ie, low walkability, high green space) for older adults that can be intervened upon.

### Limitations and strengths

This study has several limitations. First, the conceptualization of walkability and green space exposures was influenced by the availability of objectively measured data. Walkability was conceptualized using “diversity, density, and design,”^37^ which has been used extensively in past research. Additional measures of walkability, including microscale measures (eg, length of crosswalks, uneven walking surfaces, traffic speed, or number of bumps/cracks/potholes on walkways), must be studied to understand the relationship between walkability and fracture in this population.^46^ However, microscale walkability measures may also be less reliable walkability measures and difficult to collect on large scales across diverse U.S. locations. Relatedly, land use data were unavailable for this sample, so diversity of land cover captured exposure to developed or vegetative land cover types. Land cover does not capture important land uses that may facilitate walking, but land cover is related to outdoor walking among older adults.^47^ The current green space indicator is also widely used in research on green space and health,^25^ but more specific green space measures that capture parks and open spaces that can be specifically used for recreation should be examined in the future. Another limitation was that most fractures were self-reported. The validity (ie, agreement with medical records) of self-report of fractures varies by anatomical site in the WHI, being higher for hip (78%) and forearm or wrist (81%) fractures compared to spine fractures (51%), and fractures were confirmed using medical records.^48^ Importantly, participants did not report information regarding circumstances of the fracture. While this limits our ability to know if the fracture occurred outdoors or indoors, reporting fracture circumstances often suffers from recall bias and the outdoor environment may impact fracture risk (by decreasing activity levels) even if the fracture occurs indoors.^49^ Next, individual-level measures, such as low physical functioning and walking level, were self-reported at baseline, which may not capture changes to these characteristics and behaviors over time. In addition, while measures were taken to ensure representation of different racial and ethnic groups and rural residents, most of the WHI sample identified as Non-Hispanic and White and lived in urban locations. Finally, the geocoding process was conducted using ArcGIS 10.2.2 software, which has known limitations in handling certain address formats, especially those with incomplete or ambiguous address components, and remote locations that may not be well represented in the geocoding reference databases.^50^

This study also had several strengths. Our research employed WHI data to investigate outdoor and environmental risk factors for fracture in postmenopausal women, a sample well-characterized on personal-level risk factors (eg, age, race, ethnicity), with large national reach (24 states, 40 regions) and large sample size (162 000 participants) available to produce risk estimates.^32^ Current research on environmental determinants of fracture is largely limited to studies outside the United States,^12^ has not focused on large cohorts of older women residing in community settings, and does not adequately account for many known person-level risk factors.^11–15^ Compared to existing research on this topic,^11–15^^,^^21^ this study includes more diverse study participants from different racial and ethnic groups across the United States. Lastly, this study examined the relationship between NSES, walkability, and green space longitudinally to incident fractures. This is important for establishing a temporal relationship between these understudied environmental exposures and this important clinical outcome. Very few past studies have used a prospective study design to examine the relationships between neighborhood supports,^14^^,^^21^ which inevitably change over time, and fracture outcomes that may inhibit the ability of older women to *age in place.*

## Conclusion

Findings indicated that NSES and green space were associated with increased incident fractures, while walkability had a mixed association. This is a substantial contribution to the literature on risk factors for fracture among older women. Older adults and postmenopausal women are often dependent on their immediate environments as they age, and neighborhood environment accessibility is crucial to maintaining PA and independence. Uncovering the relationships between outdoor and neighborhood environments and fracture outcomes in the United States is important for encouraging active and independent aging among women*.*

## Data Availability

The data underlying this article were provided by the Women’s Health Initiative by permission. Data will be shared on request to the Women’s Health Initiative.

## Clinical trial registration

ClinicalTrials.gov: NCT00000611.
